# CO_2_ concentration forecasting in smart cities using a hybrid ARIMA–TFT model on multivariate time series IoT data

**DOI:** 10.1038/s41598-023-42346-0

**Published:** 2023-10-12

**Authors:** Pantelis Linardatos, Vasilis Papastefanopoulos, Theodor Panagiotakopoulos, Sotiris Kotsiantis

**Affiliations:** 1 Department of Mathematics, University of Patras, 265 04 Patras, Greece; 2 School of Science and Technology, Hellenic Open University, 263 35 Patras, Greece; 3 School of Business, University of Nicosia, 2417 Nicosia, Cyprus

**Keywords:** Engineering, Environmental impact

## Abstract

Carbon Dioxide (CO$_{2}$) is a significant contributor to greenhouse gas emissions and one of the main drivers behind global warming and climate change. In spite of the global economic slowdown due to the COVID-19 pandemic, the global average atmospheric CO$_{2}$ concentration reached a new record high in 2020 with its year-on-year increase being the fifth highest annual increase in 63 years, according to the National Oceanic and Atmospheric Administration. Furthermore, the years 2020 and 2019 were respectively the second and third warmest, while the decade 2010–2019 was the warmest decade ever recorded. In an attempt to curb this climate emergency, many countries and organizations globally have adopted ambitious goals and announced plans to help dramatically reduce CO$_{2}$ emissions. As part of these plans, various innovative smart city projects are being developed, focusing on implementing Internet of Things (IoT) technologies. By collecting sensor-based data, such technologies aim towards automating data-driven decision-making around carbon emission management and reduction. In this work, a hybrid machine learning system, aimed at forecasting CO$_2$ concentration levels in a smart city environment was developed using a multivariate time series dataset containing IoT sensor measurements of CO$_{2}$, as well as various environmental factors, taken at every second. The proposed system demonstrated superior performance to similar methods, while also maintaining a high degree of interpretability. More specifically, the approach was empirically compared against other similar approaches in several scenarios and use cases, thus also offering more insight into the predictive capabilities of such state-of-the-art systems. For this comparison, both traditional time series and deep learning approaches were employed, including the current state-of-the-art architectures, such as attention-based, transformer networks. Results demonstrated that, when measured across various settings and metrics, including three different forecasting horizons, the hybrid solution achieved the best overall results, and in some cases, the difference in performance was statistically significant. At the same time, insights from the system’s inner workings were extracted, shedding light on the reasoning behind the model’s predictions and the factors that contribute to them, thus showcasing its transparency. Lastly, throughout the experiments, deep learning approaches illustrated their ability to better handle the multivariate nature of the dataset and in general tended to outperform the traditional time series methods, especially for longer forecasting horizons.

## Introduction

Carbon dioxide (CO$_{2}$) levels and other greenhouse gases in the atmosphere have risen to new highs in recent years. Being one of the main drivers behind global warming, CO$_{2}$ emissions have resulted in some of the warmest years on record; in fact, the decade 2010–2019 was the warmest ever recorded. Recent predictions for the World Meteorological Organization by a team from 11 forecast centers have shown that there’s a 48% chance the globe will temporarily reach a yearly average increase of 1.5 $^{\circ }$C above pre-industrial levels of the late 1800s, between 2022 and 2026. There is consensus among scientists that such an increase, if sustained long-term, risks unleashing severe climate change effects on people, wildlife, and ecosystems—some of which may be irreversible^1^.

As a result, the Paris Agreement, adopted in 2015, aimed to strengthen the global response to the threat of climate change by keeping a global temperature rise this century well below 2 $^{\circ }$C above pre-industrial levels and given the grave risks, to strive for the 1.5 $^{\circ }$C threshold. To achieve this outcome, the agreement lays out plans to strengthen the ability of countries to deal with the impacts of climate change and maintain environmental sustainability, through appropriate financial flows, a new technology framework and an enhanced capacity-building framework^2^. One of the main goals of the agreement is for countries to bear the responsibility of reducing their CO$_{2}$ emissions. More specifically, hitting the ambitious 1.5 $^{\circ }$C mark requires almost halving global CO$_{2}$ emissions from 2010 levels by 2030 and cutting them to net zero by 2050. Implementation of the Paris Agreement is also essential for the achievement of the Sustainable Development Goals set out by the UN^3^.

Thus, in light of the increasingly serious, CO$_{2}$ emissions-derived problems, the significance of CO$_{2}$ levels monitoring and forecasting has been acknowledged, as an important means of enacting and applying appropriate proactive and reactive measures. To this end, more and more smart-city projects are employing Internet of Things (IoT) systems to enable continuous environmental monitoring by measuring CO$_{2}$ concentrations among other pollutants in urban living environments. Recent advances in IoT technologies and devices have led to the deployment of monitoring systems based on low-cost Wireless Sensor Networks (WSNs) for real-time collection of CO$_{2}$ data both in open city environments^4^ and indoor contexts^5^. Such systems seek to provide efficiencies in areas such as energy consumption^6^, transportation^7^, and more by utilizing micro-sensors, micro-controllers, wireless communication technologies, cloud IoT platforms, and advanced data analysis methods for collecting, processing and storing data, as well as for producing forecasts and visualizations to end users.

In this study, a multivariate dataset containing IoT sensor measurements of several environmental factors was used to develop a hybrid time series model, aimed at forecasting CO$_{2}$ concentration in a smart-city environment. More specifically, the approach consists of a statistical method, AutoRegressive Integrated Moving Average method (ARIMA)^8^ and a deep learning method, Temporal Fusion Transformers (TFT)^9^. c. That said, studies using time series models along with IoT technologies for emissions forecasting are relatively sparse in the literature. At the same time, the proposed system is highly interpretable by nature, meaning that the reasons behind its forecasts can be attributed back to its inputs—a critical and much-desired property for the adoption of such systems in real-life applications^10^. The developed hybrid approach was benchmarked against its separate core components, ARIMA and TFT, as well as several other methods, namely Exponential Smoothing^11^, Fast Fourier Transform (FFT)^12^, the Theta method, DeepAR^13^, N-BEATS^14^, Transformer Networks^15^, Temporal Convolutional Networks (TCN)^16^ and Long Short-Term (LSTM) Networks^17^.

The rest of this paper is organized as follows: in section “Related work”, a number of related studies pertaining to CO$_2$ emissions forecasting are presented. Subsequently, in section “Methods”, the dataset and its preprocessing are described, the proposed hybrid solution and the motivation behind it are analyzed and the implementation details around the experimental procedure are outlined. Later, in section “Results and discussion”, the results are reported and discussed. Finally, the concluding remarks of this study are provided in section “Conclusion”.

## Related work

In this section, scientific work related to this study, regarding CO$_2$ concentration and/or CO$_2$ emissions prediction, is presented. In general, CO$_2$ forecasting models can be split into four broad categories: statistical time series models, traditional machine learning models, deep learning time series models, and lastly hybrid models, which combine models from one or more of the above categories.

Statistical time series techniques are used to model data that come in the form of sequences. In the domain of CO$_2$ forecasting, this can be a series of CO$_2$ measurements captured at successive, equally spaced points in time, for example, the CO$_2$ concentration around a certain area every minute or some other time interval. Such models, including but not limited to autoregressive (AR), moving average (MA)^8^, exponential smoothing^11,18^ and structural time series models^19,20^ can naturally handle the sequential nature of the data. Among the statistical time series methods, ARIMA seems to be the most popular for CO$_2$ emissions forecasting^21,22^. That said, many others, such as ARMA^23,24^, ARIMAX^25^, NARX^26^, SARIMAX^27^ the Holt-Winters exponential smoothing model^22,27,28^ as well as the more recent Prophet model^29^ have been also applied to the same problem. Statistical time series models for CO$_2$ forecasting typically come with two main limitations. Firstly, they usually make strong assumptions about the underlying data properties, their distribution as well as time dependencies. If not satisfied, these assumptions can pose limitations on modeling, which in turn could potentially hinder performance. Secondly, in order to perform well, they often rely on human domain knowledge and expertise, which might be hard to obtain.

Traditional machine learning models have demonstrated the ability to learn complex relationships from the data itself, with little or no human intervention^30^. Such models have appeared in numerous CO$_2$ emissions forecasting studies, with linear regression (LR)^31,32^, support vector machines^27,32^, random forest (RF)^27,32,33^ and feed-forward neural networks (ANNs)^22,34^ being the most commonly used approaches. Other supervised regression approaches have also been applied, albeit to a lesser degree; these include ridge regression (RR)^32,35^, polynomial regression^36^, k-nearest neighbours^32^, extreme learning machine^23,37^, decision trees (DT)^32,35^, gradient boosting (GB)^32,35^, Gaussian processes^38^ as well as neuro-fuzzy rules (^39^ and evolutionary algorithms^40^. A great limitation of traditional machine learning models for CO$_2$ forecasting is that, unlike statistical time series methods, they cannot inherently handle the temporal nature of time series data. Firstly, they often assume that data points are independent and identically distributed (i.i.d.). However, in time series data, observations are highly correlated and exhibit complex temporal dependencies. Secondly, time series data often exhibit time-related characteristics, such as seasonality and trend. Traditional machine learning models typically struggle to effectively capture and model these patterns without additional preprocessing steps such as data transformations and feature engineering.

Deep learning time series models, similarly to statistical time series methods, can inherently handle sequential data^41^. However, instead of relying on human expertise to guide them, they allow for complex data relationships, including temporal ones, to be learned directly from the data itself. More specifically, such models have been shown to be particularly efficient in learning high-level data representations arising from complex variable relationships^42^. Deep learning models for time series usually make use of one or more of the following neural network architectures as their building blocks: recurrent neural networks^43^, convolutional neural networks^44^ and more recently attention-based neural networks, such as transformers^15^. Compared to other methods, deep learning-based approaches, either pure or hybrid, for CO$_2$ emissions forecasting are much less common in literature. Most approaches employ recurrent neural networks^45^ and their variations, such as long short-term memory networks^46–48^ and bidirectional long short-term memory networks^49^, while attention-based recurrent neural networks have also been applied^50^. Deep learning models can be very powerful but do not come without their own drawbacks. Due to their superior expressiveness, they are prone to overfitting^51^ and often require large amounts of training data to generalize well. Furthermore, the majority of deep learning models suffer from interpretability issues, as they often act as black boxes and their decisions cannot be explained well^52^.

To address the shortcomings of each of the previously discussed single-model categories, hybrid approaches that combine one or more models from one or more categories are often used. Specifically for CO$_2$ emissions forecasting, a framework integrating index decomposition analysis (IDA) along with ANNs and data envelopment analysis (DEA)for the modeling greenhouse gases produced annually by Canada’s industrial sector was employed in^53^, while a model combining a general regression neural network and scenario analysis was constructed in^54^, in order to forecast China’s carbon emissions between 2016 and 2040, under different scenarios, based on various influencing factors. In^55^, a pure deep-learning hybrid was proposed as long short-term networks were combined with convolutional neural networks to predict CO$_2$ levels for the year 2020. In an older study^56^, an ensemble Adaptive Neuro-Fuzzy Inference System (ANFIS) learning method, integrating the advantages of both fuzzy inference systems and ANNs was applied to predict CO$_2$ emissions. Furthermore, in^57^, a hybrid approach blending the results of nine different algorithms (a variety of machine learning, statistical time series, and deep learning) along with mathematical programming was developed to forecast the emission rate of greenhouse gases in Iran between 2018 and 2028, while in^58^ two hybrids were proposed; the first combined the metabolic non-linear grey model (MNGM) with ARIMA, while the second fused MNGM with a back propagation neural network model (BPNN). Both models were applied to predict the carbon emission trajectory of China, the US, and India for the 2019–2030 period. A two-step hybrid method was developed in^59^: first, CO$_2$ emissions are predicted using multiple regression, Gaussian process regression, and ANN models separately, whose predictions are then fed as input to an ANN to produce the final prediction. Three different models, namely ANNs, RF, and particle swarm optimization (PSO) were integrated into a single approach in^60^ to make projections regarding the Chinese commercial sector CO$_2$ emissions from 1997 to 2017. Although a number of recent studies have developed hybrid approaches to the problem of CO$_2$ forecasting, existing hybrid solutions do not focus on the benefits of using attention mechanisms, such as TFT, in time series, which have been shown to achieve both improved performance over comparable recurrent networks and a greater degree of interpretability through attention weights^9^. More specifically, the capabilities of TFT were demonstrated in^61^, where Huy et al. combined it with linear regression for Short-Term Electricity Load Forecasting. Their hybrid approach was compared against a number of models both statistical and deep learning ones, demonstrating its superiority. Similar results were reported in^62^, where TFT-based hybrids outperformed other comparable models in the vast majority of performance metrics for wind speed forecasting. At the same time, the model’s interpretability was illustrated, offering insights into the deciding factors of wind speed forecasts during each season (Spring, Summer, Autumn, and Winter). The interpretable nature of TFT was further illustrated in^63^, where TFT was applied to a multivariate dataset, including historical tourism volumes, travel forum, and search engine data as well as monthly new confirmed cases of travel destinations to forecast tourist volumes amid the COVID-19 pandemic. Analysis revealed, among other insights, to what degree pandemic-related search engine data affected traveling volumes.

**Figure 1:**
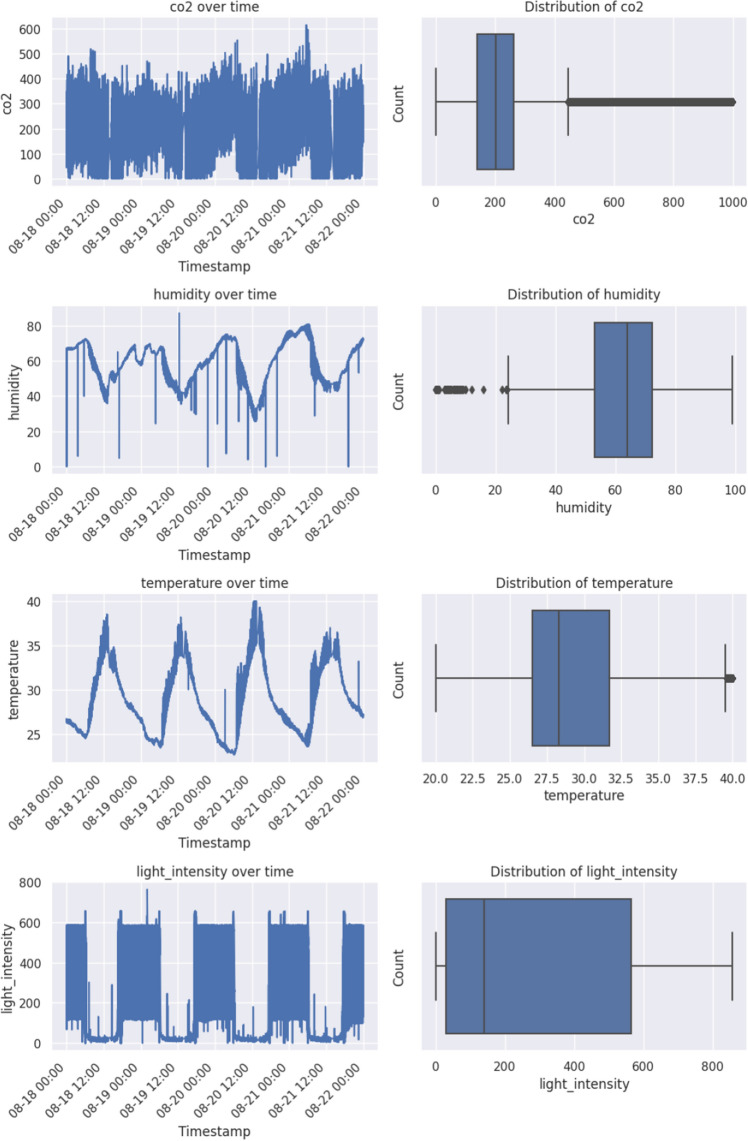
Raw data distribution for each variable over a 3-day period (left). Box-plot for each variable over the entire 3-month period (right).

## Methods

### Data description and preprocessing

For both modeling and evaluation purposes a multivariate CO$_2$ dataset, originally proposed in^35^, containing measurements observed by a WSN was used. More specifically, the network consisted of three main parts including the sensor device, the sink node device, and the server. All IoT devices were deployed to gather measurements regarding environmental conditions every 1 s for 24 h per day over a three-month period. In particular, the following environmental factors were recorded: CO$_2$ concentration in ppm (part per million), the temperature in $^{\circ }$C, light intensity in foot candles (approx. 0–1000), and percentage of humidity. Table 1 contains a summary of the dataset. Furthermore, on the left-hand side of Fig. 1, the raw time series of each variable is depicted over a 3-day period, while on the right-hand side, the entire data distribution of each variable is shown in the form of a box-plot. As mentioned, more information about the dataset and its collection process can be found in^35^.

**Table 1:** Summary of CO$_2$ dataset.

Environmental factor	Unit	Total records	Bad records	Empty records	Value-range
CO$_2$ concentration	ppt	6114708	0	32	0–1000
Temperature	$^{\circ }$C	6114708	9	0	20–40
Humidity	%	6114708	3	0	0–100
Light Intensity	foot-candles	6114708	0	40	0–1000

For each environmental factor, there are measurements from three different IoT sensors.

In terms of data preprocessing, firstly, all duplicated values were removed from the dataset. Subsequently, any bad records, i.e. records that did not adhere to the dataset schema, were dropped. The next step included some basic outlier handling by capping values found to be outside reasonable bounds—most likely caused by some software or hardware bug. More specifically, the following ranges were used to restrict the values of each column: 0–1000 for CO$_2$ concentration, 20–40 for temperature, 0–100 for humidity, and 0–1000 for light intensity. These values were determined based on the data distributions, boxplots for which are presented in Fig. 1. Any values outside these ranges represent the most extreme 0.3% or less of data points for any of the variables. This process resulted in 19,492 values being clipped in order to be kept within the aforementioned ranges, which can be broken down as follows: 18162 CO$_2$ concentration values, 582 humidity values, 374 temperature values, and 374 light intensity values.

The deployment of three IoT sensors meant that for each point in time, in this case for every second, there were typically three different values for each environmental factor, each corresponding to a separate sensor. Such large data volumes, combined with limited computational resources, resulted in the values of the IoT data being averaged at an hourly level for modeling and evaluation purposes. That is, for every column, any data points, from any sensor, present in a given hour of the day (1–24) were averaged to form a single data point in time corresponding to the hourly period in question. It is also worth noting that any empty values present in the data were ignored during this calculation. Lastly, in the cases where, after performing this averaging for a given hour (1–24) in a day, the result was an empty value, i.e. all second-level measurements from all three sensors for that hour had empty values, then that hour was completely ignored.

### Proposed hybrid time series approach

This section outlines the motivation behind the proposed hybrid approach and its two main blocks, AutoRegressive Integrated Moving Average (ARIMA) and Temporal Fusion Transformer (TFT), are analyzed in detail.

#### Motivation

When it comes to the application of machine learning systems to smart cities, although several studies were conducted over the last decade, the number of those focusing specifically on the CO$_2$ emissions prediction issue with IoT technologies is limited, compared to other applications^64,65^. An older study^66^, developed three different machine learning models, Naive Bayes, ANN, and DT, using sensor information to forecast CO$_2$ levels, as a proxy of air quality in smart environments. In a more recent study^67^, the RF algorithm was applied to estimate the CO$_2$ content in the air of a smart home, based on factors such as the internal and external temperature, internal relative humidity, and the date and time of day, while in^35^ a variety of models, namely RF, GB, LR, RR, DT and LSTM were developed and then compared for CO$_2$ concentration estimation. More studies were conducted over the last two years: in 2021, a system that utilized real-time in-vehicle sensor data was proposed^48^, in order to forecast the vehicle’s CO$_2$ levels using an LSTM network; then in 2022, a multi-linear regression approach was adopted^68^ to predict CO$_2$ emissions, based on IoT traffic flow data, taking into account both congested and uncongested conditions.

In this work, a new, highly interpretable, hybrid model, able to naturally handle time series data (unlike traditional machine learning models, such as RF, DT, LR, GB, etc), was built for CO$_2$ concentration forecasting. Hybrid approaches have a long history in time series forecasting^69^. One of the first and most influential works on hybrid models for time series was that of Zhang^70^, combining ARIMA with a neural network model. Since then, hybrid models for time series have gained a lot of popularity and a wide variety of combinations have been proposed^71–73^.

The fundamental motivation behind hybrid methods is that the combination of the best of statistical and machine learning methods would bring out the best of both worlds and counter-balance the limitations of each approach using the strengths of the other. Another argument in favor of hybrid approaches is that although deep learning methods have exhibited extraordinary results in domains such as natural language and computer vision, they have not yet delivered on their promise when it comes to time series forecasting. This was demonstrated in the M4 competition^74^, where various time series models were benchmarked across 100,000 time-series datasets. The results of the study showed, among other things, that 1. the top-performing approaches involved combinations of models, 2. the least-performing approaches were either pure statistical or pure machine learning models, 3. the best overall approach was a hybrid solution that utilized both statistical and machine learning features and 4. increased model complexity potentially leads to enhanced forecasting performance. Lastly, approaches purely based on deep learning methods usually act as black boxes, making their output hard to interpret^10^, which makes simpler approaches often more desirable in real-life applications, especially if the loss in performance is not significant.

The most common and widely used hybrid combination is the one comprised of ARIMA and artificial neural networks^69^, highlighting the versatility of both methods in a variety of time series domains. This study builds on this well-established trend, by combining ARIMA and TFT, a neural-network-based architecture, as its two main blocks for CO$_2$ concentration forecasting. The importance of incorporating TFT is twofold: firstly, when benchmarked against other comparable deep learning approaches, such as simple LSTMs, TFT has demonstrated improved performance in various time series forecasting tasks^61,62^, and secondly, through the analysis of its attention weights it can achieve a greater degree of interpretability, thus alleviating black-box concerns^9,62,63^.

#### Step 1: ARIMA forecasting

The first step of the proposed pipeline is to forecast CO$_2$ concentration values using an ARIMA model. ARIMA models cannot handle multivariate time series data and therefore only past values of the target variable (CO$_2$ concentration) were used for modeling. As a result, any forecasts from this step leave out important additional information from other environmental factors present in the dataset, detailed in section “Data description and preprocessing”, such as humidity, temperature, and light intensity. On the other hand, by focusing purely on the previous values of CO$_2$, this step makes sure that such information–arguably the most important piece of information—is more heavily weighted, once the models’ outputs are combined in step 3.

The ARIMA^9^ family of models, is one of the most popular and effective statistical models for time series forecasting. It is based on the fundamental principle that the future values of a time series are generated from a linear function of past observations and error terms. ARIMA consists of three sub-components: “AR”, “I” and “MA”; in this section, these sub-parts are analyzed in deeper detail.

The “AR” component, standing for “AutoRegressive” indicates that the target variable is modeled as a linear combination of its past values. At time *t* the target variable’s value $y_t$ can be mathematically expressed by Eq. (1):

1 $$\begin{aligned} y_t = \beta _0 + \beta _1 y_{t-1} + \beta _2 y_{t-2}+ \ldots +\beta _p y_{t-p} +  \varepsilon   _t, \end{aligned}$$

where $y_{t-1}, y_{t-1} \ldots y_{t-p}$ are the target variable’s past values at time-steps $t-1, t-2,\ldots , t-p$, $\beta _0, \beta _1 \ldots \beta _p$ are the parameters of the regression and $\epsilon _t$ is white noise at time *t*. The parameter *p* represents the maximum lag, also known as the lag order; for example, a model notated as AR(p) denotes an AutoRegressive model of order p.

The “I” element, stands for “Integrated”, denoting that a differencing step to the modeled data. Differencing is a method of transforming a time series dataset to make it stationary, i.e. remove its time-related properties, such as trend and seasonality. It is conducted by subtracting the past observation from the current observation and can be repeated as many times as needed, i.e. differencing the differences themselves, until all temporal dependencies have been eliminated. The term “difference order” refers to the number of times differencing has been performed. The reason for differencing is that differences are more stationary than raw, un-differenced values. As a result, the statistical properties of the produced model are unaffected by the period the sample was taken. In general, models based on stationary data are more reliable. Assuming a first-order differencing has taken place, Eq. (1) can be re-written, as shown in Eq. (2):

2 $$\begin{aligned} \begin{aligned} y_t - y_{t-1}&= \beta _0 \\ {}&\quad + \beta _1 (y_{t-1} - y_{t-2}) \\ {}&\quad + \beta _2 (y_{t-2} - y_{t-3}) \\ {}&\quad + \ldots \\ {}&\quad + \beta _p (y_{t-p} - y_{t-p-1}) \\ {}&\quad + \varepsilon _t \end{aligned} \end{aligned}$$

The order of differencing is controlled by a parameter, usually denoted by the letter “d”.

The “MA” part, standing for “Moving Average”, refers to a model where the target variable only depends on the previous forecast errors. This means that a Moving Average model forecasts the target variable as a linear combination of the errors of its own forecasts regarding past time steps, also called residuals. According to a Moving Average model, at time *t* the target variable’s value $y_t$ can be mathematically described by Eq. (3):

3 $$\begin{aligned} y_t = \theta _0 + \theta _1 \varepsilon _{t-1} + \theta _2 \varepsilon _{t-2}+ \ldots +\theta _p \varepsilon _{t-q} + c, \end{aligned}$$

where $\epsilon _{t-1}, \varepsilon _{t-1} \ldots \varepsilon _{t-q}$ represent the errors in the model’s forecasts at time-steps $t-1, t-2,\ldots , t-q$ respectively, $\theta _0, \theta _1 \ldots \theta _q$ are the parameters of the regression and *c* is the mean of the series value. The parameter *q* controls the number of previous errors to consider when forecasting the next time step; a model notated as MA(q) denotes a Moving Average model of order q.

In summary, an ARIMA(p,d,q) model uses a combination of the “AR” and MA” models, whose orders are controlled by the p and q parameters respectively. This model mixture, along with integrated differencing (“I”), the order of which is determined by d, allow for powerful time series analysis.

#### Step 2: TFT Forecasting

This step attempts to address the main shortcomings of ARIMA, used in step 1. Although ARIMA is a powerful tool for time series forecasting, due to its linearity and stationarity assumptions, it is not as effective in modeling more complex data relationships, often encountered in real-world time series. Furthermore, to produce forecasts for a given time series, it can only learn from that series’ past values alone, being unable to incorporate and learn from external time series, also known as covariates.

Newer deep learning models, such as TFT, address these shortcomings as they are able to model multivariate time series data and extract valuable information with regard to how these different variables interact with one another. As a result, any forecasts produced in this step take into account both the past values of the target variable (CO$_2$ concentration) as well as all the available external environmental factors present in the dataset, namely temperature, humidity, and light intensity.

**Figure 2:**
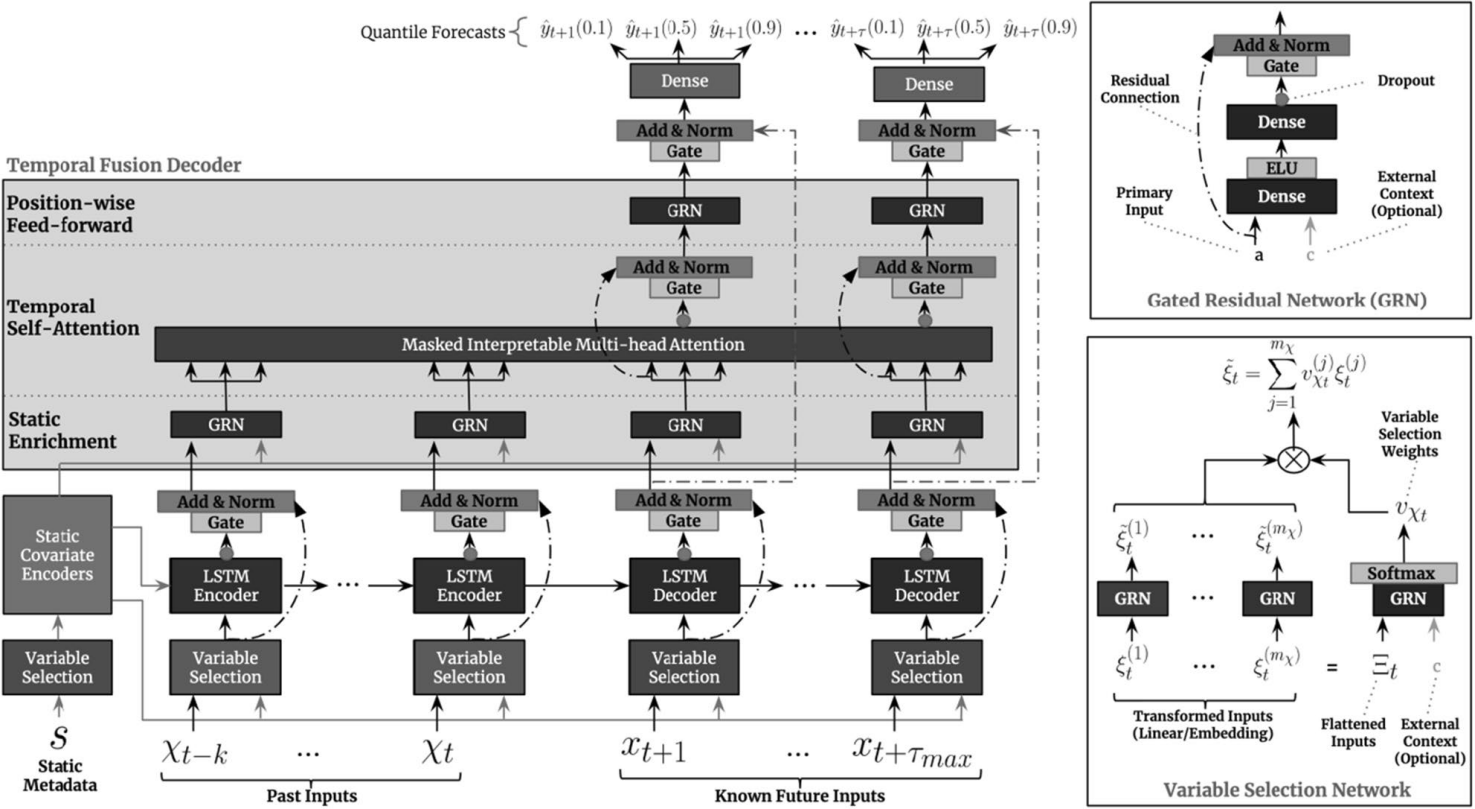
TFT Model architecture. Adapted from^9^.

TFT is a state-of-the-art deep learning model purposefully designed for time series modeling. Its complex architecture, displayed in Fig. 2, consisting of LSTM encoding layers and interpretable transformer attention layers, offers more features and capabilities than any of the previously proposed deep learning-based architectures. The five components that make up the basic structure of the TFT are analyzed below:

1. *Gating mechanisms*: It is often challenging to gauge the degree of necessary non-linear processing needed, and there may be circumstances in which simpler models are advantageous, such as when datasets are not big enough or contain a lot of noise. TFT alleviates both these issues by employing Gated Residual Networks (GRN)^75^. A GRN accepts a primary input $\alpha$ and an optional context vector **c** and its output is shown in Eqs. (4, 5 and 6):
  4 $$\begin{aligned}&\text {GRN}_{\omega }(a, c) = \text {LayerNorm} (a + GLU_{\omega }(\eta _{1})), \end{aligned}$$
  5 $$\begin{aligned}&\eta _{1} = W_{1,\omega } \eta _{2} + b_{1,\omega },\end{aligned}$$
  6 $$\begin{aligned}&\eta _{2} = \text {ELU} (W_{2,\omega } \alpha + W_{3,\omega }c + b_{2,\omega }), \end{aligned}$$
  where ELU is the Exponential Linear Unit activation function^76^, $\eta _{1} \in {\mathbb {R}}^{d_{model}}, \eta _{2} \in {\mathbb {R}}^{d_{model}}$ are intermediate layers, LayerNorm is the standard layer normalization of^77^, and $\omega$ is a weight-sharing index. Additionally, Gated Linear Units (GLUs)^78^, which gating layers are built on, allow for skipping over any components that are not helpful for modeling a particular dataset. The output of a GLU accepting $\gamma \in {\mathbb {R}}^{d_{model}}$ as its input can be expressed as shown in Eq. (7):
  7 $$\begin{aligned} \text {GLU}_{\omega }(\gamma ) = \sigma (W_{4, \omega } \gamma + b_{4, \omega }) \odot (W_{5, \omega } \gamma + b_{5, \omega }), \end{aligned}$$
  where $\sigma$ is the sigmoid activation function, $W \in {\mathbb {R}}^{d_{model} {X} d_{model}}$ is the set of weights, $b \in {\mathbb {R}}^{d_{model}}$ is the set of biases, $\odot$ is the element-wise Hadamard product, and $d_{model}$ hidden state size, which is common across the whole architecture. In summary, GLU enables TFT to regulate how much the GRN contributes to the initial input $\alpha$. In the extreme case, it is able to even skip a layer entirely if necessary, thus zeroing out any nonlinear effects.
2. *Variable selection networks*: It is often challenging to pre-determine which factors going to be important for a given dataset/problem. To address this, TFT offers out-of-the-box instance-wise feature selection for both static and time-dependent covariates. Not only do variable selection networks shed light on which features contribute mostly to TFT’s forecasts, but also enable TFT to get rid of any noisy inputs that could potentially hamper its performance.
  TFT calculates variable selection weights as described in Eq. (8):
  8 $$\begin{aligned} \upsilon _{\chi _{t}} = \text {Softmax}(\text {GRN}_{\upsilon _{\chi }}(\Xi _{t}, \text {c}_{s})), \end{aligned}$$
  where $\Xi _{t} = {\left[ {\xi _{t}^{(1)}}^\intercal ,\ldots ,{\xi _{t}^{(\mu _{\chi })}}^\intercal \right] }^\intercal$ the flattened array of all past datapoints at time t, $\xi _{t}^{(j)} \in {\mathbb {R}}^{d_{model}}$ the transformed input of the *j*-th variable at time *t*, c$_{s}$ a context vector, computed by a static covariate encoder. Each $\xi _{t}^{(j)}$ is also passed through its own GRN, as shown in Eq. (9), producing another non-linear transformation ${\tilde{\xi }}^{(j)}_{t}$.
  9 $$\begin{aligned} {\tilde{\xi }}^{(j)}_{t} = \text {GRN}_{{\tilde{\xi }}_{(j)}} (\xi _{t}^{(j)}) \end{aligned}$$
  These transformations are then weighted to produce the final contribution as shown in Eq. (10):
  10 $$\begin{aligned} {\tilde{\xi }}_{t} = \sum _{j=1}^{\mu _{\chi }} \upsilon _{\chi _{t}}^{(j)} {\tilde{\xi }}^{(j)}_{t}, \end{aligned}$$
  where $\upsilon _{\chi _{t}}^{(j)}$ is the *j*-th element of vector $\upsilon _{\chi _{t}}$.
3. *Static covariate encodings*: By design, TFT pays special attention to static metadata by creating four different context vectors. More specifically, these are contexts regarding the local processing of temporal information, the enhancement of temporal information with static features, as well as temporal feature selection. These context vectors, produced by different GRN encoders, are strategically placed in different parts of the architecture where the impact of static variables can prove significant.
4. *Temporal processing*: To both improve the model’s interpretability and better understand the long-term relationships between various time steps, TFT adjusts the underlying transformer network’s multi-head attention mechanism. Just like any typical attention mechanism, it calculates values $V \in {\mathbb {R}}^{N \times d_{v}}$ based on the relationships between keys $K \in {\mathbb {R}}^{N \times d_{\text {attention}}}$ and queries $Q \in {\mathbb {R}}^{N \times d_{\text {attention}}}$ as shown in Eq. (11):
  11 $$\begin{aligned} \text {Attention}(Q, K, V) = A(Q,K)V, \end{aligned}$$
  where $A(Q,K) = \text {Softmax}(\frac{QK_T}{\sqrt{d_{\text {attention}}}})$. Multi-head attention uses different attention heads for different representation spaces, which are mathematically represented by Eq. (12)
  12 $$\begin{aligned} \text {Multihead}(Q, K, V) = [H_1,\ldots ,Hm_H]W_H \end{aligned},$$
  where $H_h = \text {Attention}(QW_Q^{(h)},KW_K^{(h)}VW_V^{(h)})$ and $W_Q^{(h)} \in {\mathbb {R}}^{d_{\text {model}} \times d_{\text {attention}}}$, $W_K^{(h)} \in {\mathbb {R}}^{d_{\text {model}} \times d_{\text {attention}}}$, $W_V^{(h)} \in {\mathbb {R}}^{d_{\text {model}} \times d_V}$ are head-specific weights for queries, keys, and values respectively, while the outputs of all heads $H_h$ are linearly combined by the weights $W_H \in {\mathbb {R}}^{(m_H \dot{d}_V) \times d_{\text {model}}}$. Since in each head, different values are calculated, attention weights cannot adequately explain the contribution of a particular variable. To this end, TFT adjusts its multi-head attention mechanism to share values in each head. The equations are then modified as shown in Eq. (13):
  13 $$\begin{aligned} \begin{aligned} {\tilde{H}}&= {\tilde{A}}(Q,K)VW_V \\&= \frac{\sum _{h=1}^{m_H} A(QW_Q^{(h)}, KW_K^{(h)})}{H}VW_V \\&= \frac{\sum _{h=1}^{m_H} \text {Attention}(QW_Q^{(h)}, KW_K^{(h)}, VW_V)}{H} \end{aligned} \end{aligned},$$
  where $W_V \in {\mathbb {R}}^{d_\text {model} \times d_V}$ denotes the array of weights shared by the different heads and $W_H \in {\mathbb {R}}^{d_{\text {attention}} \times d_{\text {model}}}$ represents the final linear transformation.
5. *Predictions intervals and loss functions*: In addition to predicting single points in time, TFT also produces forecasts for whole intervals. This is accomplished by predicting different percentiles or quantiles, such as the 10th, 50th, and 90th. These interval predictions are produced simultaneously at each time step by linearly transforming a decoder’s output according to Eq. (14):
  14 $$\begin{aligned} {\hat{y}}(q,t,\tau ) = W_q {\tilde{\psi }}(t, \tau ) + b_q \end{aligned},$$
  where $W_q \in {\mathbb {R}}^{1 \times d}$, $b_q \in {\mathbb {R}}$ are the coefficients for a given quantile q

Regarding its training, TFT learns by optimizing the summed quantile loss over all quantile outputs as shown in Eq. (15):

15 $$\begin{aligned} \text {Loss}(\Omega , W) = \sum _{y_t \in \Omega } \sum _{q \in Q}\sum _{\tau =1}^{\tau _{\text {max}}} \frac{QL(y_t, {\hat{y}}(q, t -, \tau ), q)}{M \tau _{\text {max}}} \end{aligned},$$

where $QL(y, {\hat{y}}, q) = q \max (0, y-{\hat{y}}) + (1-q) \max (0, {\hat{y}} -y)$, $\Omega$ denotes the domain of training set, *W* the model’s weights, *Q* the set of quantiles e.g. $Q = \{0.1, 0.9\}$ and *M* the number of samples in the training set.

#### Step 3: Hybrid ARIMA-TFT Ensemble

**Figure 3:**
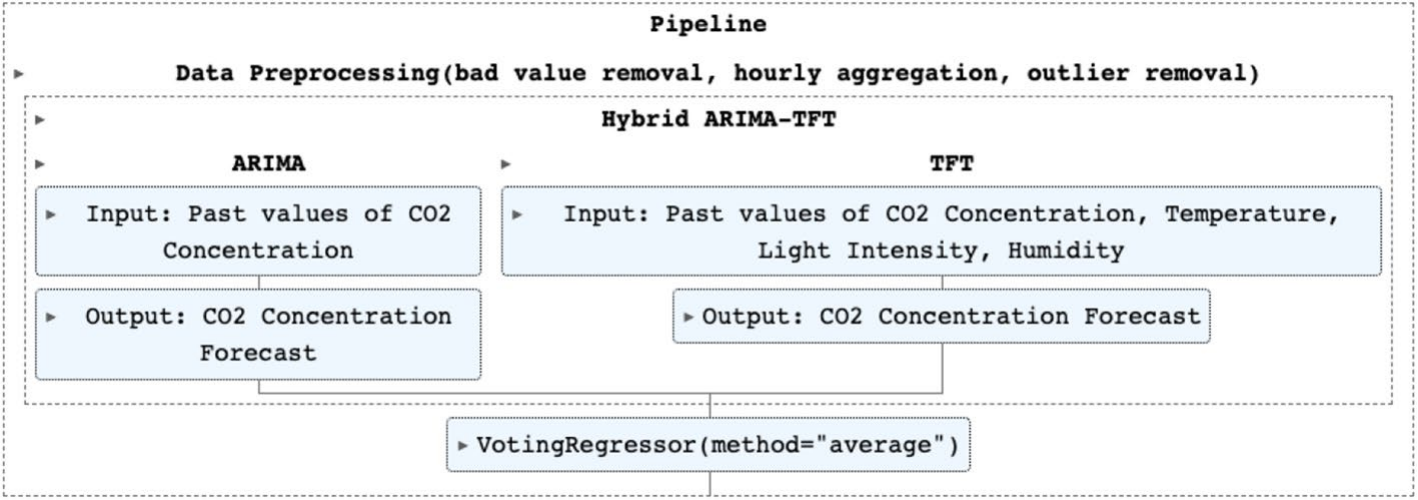
Hybrid ARIMA-TFT Pipeline.

Since ARIMA focuses exclusively on the linear relationships present in the data, it is better equipped than TFT to capture them in its forecasts. On the other hand, TFT forecasts take into account the non-linear relationships, while also taking advantage of the rich amount of extra information, thus fully exploiting the multivariate nature of the dataset. In this step, in order to use the strengths of both while balancing out their individual weaknesses, predictions from steps 1 and 2 are combined using a voting regressor ensemble, producing the average of the two independent CO$_2$ concentration forecasts. A flowchart of the whole process is presented in Fig. 3.

#### Interpretability

Interpretability refers to the degree a model’s output can be explained and attributed to the corresponding input variables. The higher the interpretability of a model, the easier it is to understand why certain predictions were made by a model, given the respective inputs the model received^79^. Interpretability is crucial and a much-desired property for all automated systems, but especially those to be adopted in sensitive yet critical domains, where human lives can be directly affected^10^, such as smart city technologies. Both sub-components of the hybrid ensemble are interpretable on their own without the need to apply any post hoc interpretability methods. More specifically, ARIMA is a so-called white box model; it’s linear and simple by design and therefore naturally interpretable. Furthermore, TFT’s architecture is transformer-based and uses a novel multi-head attention mechanism that can provide extra interpretable insights into its temporal dynamics by measuring and ranking the importance of each input variable with respect to its forecasts. As a result, the proposed hybrid ensemble is also inherently interpretable.

### Other time series models used for comparison

To assess the predictive capabilities as well as the robustness of the proposed method in different scenarios, comparisons were drawn against ten other forecasting approaches. These can be split into two broad categories: statistical and deep learning time series methods. The first category includes the AutoRegressive Integrated Moving Average (ARIMA)^8^, Exponential Smoothing^11^, Fast Fourier Transform (FFT)^12^ and Theta^80^ methods. The second category encompasses the following methods: DeepAR^13^, N-BEATS^14^, a Transformer Network^15^, Temporal Fusion Transformers (TFT)^9^, Temporal Convolutional Network (TCN)^16^ and Long Short-Term (LSTM) Network^17^. Most statistical time series models can only accept univariate data, in this case only past values of CO$_2$, ignoring other environmental variables, and therefore their relationships; in contrast, deep learning models can handle multivariate time series data and model such complex relationships. Table 2 shows what types of data are supported per model as well as information on covariates, which are discussed in greater detail in section “Past and future covariates”.

### Past and future covariates

In time series problems, a distinction is usually made between the target time series and the so-called covariate time series. The term “target time series” refers to the time series whose values are to be predicted, based on its history. For the purposes of this study, the values of CO$_2$ concentration are considered the target time series. The term “covariate time series” refers to time series, which may be useful in forecasting the target series, but no forecast is made about them.

Covariate series can be further split into past and future covariates depending on whether they can be known in advance or not. Time series whose past values are known at prediction time are referred to as past covariates. In this study, the values of temperature, humidity, and light intensity contained in the dataset are all considered past covariates. Time series whose future values are already known at prediction time is referred to as future covariates. These can represent known future information such as holidays, or even predictions such as a weather forecast, and are perfectly valid to incorporate in time series modeling when possible. In this study, the “hour of the day” (1–24), “day of the week” (1-7), “day of the month” (1-31), and “month of the year” (1-12) were all used as future covariates. Such temporal attributes can be very powerful as they allow models to better and more easily capture the trend and seasonality of the target series.

It should be noted that not all models support the use of past and future covariates. In general, simpler statistical models, such as ARIMA and Exponential Smoothing cannot deal with any type of covariates and can only accept a single target series. In comparison, the majority of deep learning methods are capable of natively handling past covariates, however, only a limited number of models can make use of future covariates. In Table 2, the types of covariates supported and used by each model in this study are presented in detail.

**Table 2:** Breakdown of the forecasting models and corresponding types of covariates used in this study.

Model	Univariate	Multivariate	Past covariates	Future covariates
ARIMA	$\checkmark$			
DeepAR		$\checkmark$		$\checkmark$
ES	$\checkmark$			
FFT	$\checkmark$			
Hybrid		$\checkmark$	$\checkmark$	$\checkmark$
LSTM		$\checkmark$	$\checkmark$	
N-BEATS		$\checkmark$	$\checkmark$	
TCN		$\checkmark$	$\checkmark$	
TFT		$\checkmark$	$\checkmark$	$\checkmark$
Theta	$\checkmark$			
Transformer		$\checkmark$	$\checkmark$	

### Experimental setup

A total of 30 cases were examined, using different data splits, forecasting horizons, training methodologies, and metrics. These cases are presented in detail in Table 3. For all the experiments, the Darts Python library^81^ was extensively utilized. The final sets of hyperparameters used to train each model, in order to reproduce the reported results can be found in Supplementary Table S1, while the full technical implementation can be found at the following public GitHub repository: https://github.com/ML-Upatras/co2-concentration-forecasting.

**Table 3:** Experimental setup outline.

Case #	Forecasting horizon	Data split (Train-Test)	Retraining	Evaluation metric
Case 1	Short-term (1 h)	80–20	Yes	RMSE
Case 2	Short-term (1 h)	80–20	No	RMSE
Case 3	Short-term (1 h)	90–10	Yes	RMSE
Case 4	Short-term (1 h)	90–10	No	RMSE
Case 5	Mid-term (24 h)	80–20	Yes	RMSE
Case 6	Mid-term (24 h)	80–20	No	RMSE
Case 7	Mid-term (24 h)	90–10	Yes	RMSE
Case 8	Mid-term (24 h)	90–10	No	RMSE
Case 9	Long-term (168 h)	80–20	Yes	RMSE
Case 10	Long-term (168 h)	80–20	No	RMSE
Case 11	Short-term (1 h)	80–20	Yes	MAE
Case 12	Short-term (1 h)	80–20	No	MAE
Case 13	Short-term (1 h)	90–10	Yes	MAE
Case 14	Short-term (1 h)	90–10	No	MAE
Case 15	Mid-term (24 h)	80–20	Yes	MAE
Case 16	Mid-term (24 h)	80–20	No	MAE
Case 17	Mid-term (24 h)	90–10	Yes	MAE
Case 18	Mid-term (24 h)	90–10	No	MAE
Case 19	Long-term (168 h)	80–20	Yes	MAE
Case 20	Long-term (168 h)	80–20	No	MAE
Case 21	Short-term (1 h)	80–20	Yes	MAPE
Case 22	Short-term (1 h)	80–20	No	MAPE
Case 23	Short-term (1 h)	90–10	Yes	MAPE
Case 24	Short-term (1 h)	90–10	No	MAPE
Case 25	Mid-term (24 h)	80–20	Yes	MAPE
Case 26	Mid-term (24 h)	80–20	No	MAPE
Case 27	Mid-term (24 h)	90–10	Yes	MAPE
Case 28	Mid-term (24 h)	90–10	No	MAPE
Case 29	Long-term (168 h)	80–20	Yes	MAPE
Case 30	Long-term (168 h)	80–20	No	MAPE

#### Lookback windows and forecasting horizons

A lookback window refers to a given period of time, preceding the time of prediction, during which the model has access to information that it learns from in order to make forecasts. On the other hand, a forecasting horizon indicates how far in the future a model predicts. In a smart city setting, predictions regarding different combinations of lookback windows and forecasting horizons can be used for different planning purposes. In this study, three different settings of lookback windows and forecasting horizons were experimented with:

- Short-term forecasting horizon: Predicting the CO$_2$ concentration after 1 h given a lookback window of 24 h.
- Mid-term forecasting horizon: Predicting the CO$_2$ concentration after 1 one day (24 h), given a lookback window of 168 h.
- Long-term forecasting horizon: Predicting the CO$_2$ concentration after 1 one week (168 h), given a lookback window of 240 h.

#### Data splits

For all experiments, the dataset was split between train and test data. The former was available to the models during the training, error-minimizing procedure, while the latter remained unknown to the models during the training process and was only used for evaluation purposes. More specifically, the experiments for the short-term and the mid-term forecasting horizons were performed for two different splits of train-test data, 90–10 and 80–20 splits. However, in the case of long-term forecasting horizon, the 90–10 split was not possible due to a lack of the required number of instances for the prediction.

#### Training methodology (retraining vs. no-retraining)

Statistical time series models are always re-trained on the entire available history, once new points in time become available, thus always expanding their look-back window. This process of retraining from scratch every time new data points are observed can be very resource-consuming, especially for deep-learning models, which are notoriously resource hungry. That said, deep learning methods do offer the alternative of only being trained once on the initial sequence and then learning from new observations in a recursive manner, without visiting the entire sequence again. In this study, all experiments regarding deep learning models were performed in both settings, and results were reported for both ways of training deep learning methods (with retraining and without).

#### Evaluation metrics

In terms of evaluation metrics, the root mean squared error (RMSE), the mean absolute error (MAE), as well as the mean absolute percentage error (MAPE), were employed to assess the performance of each time series model. Both RMSE and MAE measure the differences between predicted values and ground-truth values. It should be noted that larger errors have a disproportionately negative effect on both and, as a result, they are sensitive to outliers. Using the differences between predicted values and ground-truth values without taking into account the magnitude of the values involved, can be somewhat unintuitive since the values involved can vary hugely among the different problems. MAPE tries to solve this, by returning the error as a percentage of the original value the regressor tried to predict.

The RMSE of an estimator for a given dataset of n data points, defined as shown in Eq. (16):

16 $$\begin{aligned} {\text {RMSE}}=\sqrt{\frac{1}{n} \sum _{i=1}^n \left( y_i-\hat{y_i}\right) ^2} \end{aligned}$$

where $y_i$ is the observed value of the i-th data point and $\hat{y_i}$ the estimated values of the i-th data point

The MAE of an estimator for a given dataset of n data points, defined as shown in Eq. (17):

17 $$\begin{aligned} {\text {MAE}}=\frac{1}{n} \sum _{i=1}^n \left( |y_i-\hat{y_i}|\right) \end{aligned}$$

where $y_i$ is the observed value of the i-th data point and $\hat{y_i}$ the estimated values of the i-th data point

The MAPE of an estimator for a given dataset of n data points, defined as shown in Eq. (18):

18 $$\begin{aligned} {\text {MAPE}}={\frac{100\%}{n}}\sum _{i=1}^{n}(\left| {\frac{y_{i}-\hat{y_i}}{y_{i}}}\right| ) \end{aligned}$$

where $y_i$ is the observed value of the i-th data point and $\hat{y_i}$ the estimated values of the i-th data point

## Results and discussion

### Short-term forecasting horizon

**Figure 4:**
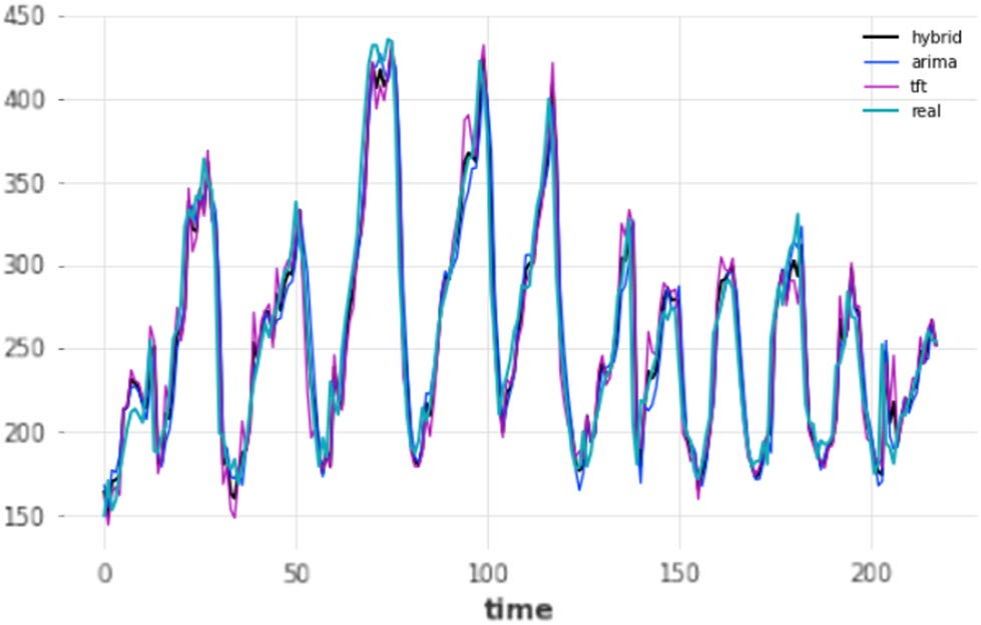
Short-term (1 h) forecasts vs ground truth for test set; split: 80–20.

**Figure 5:**
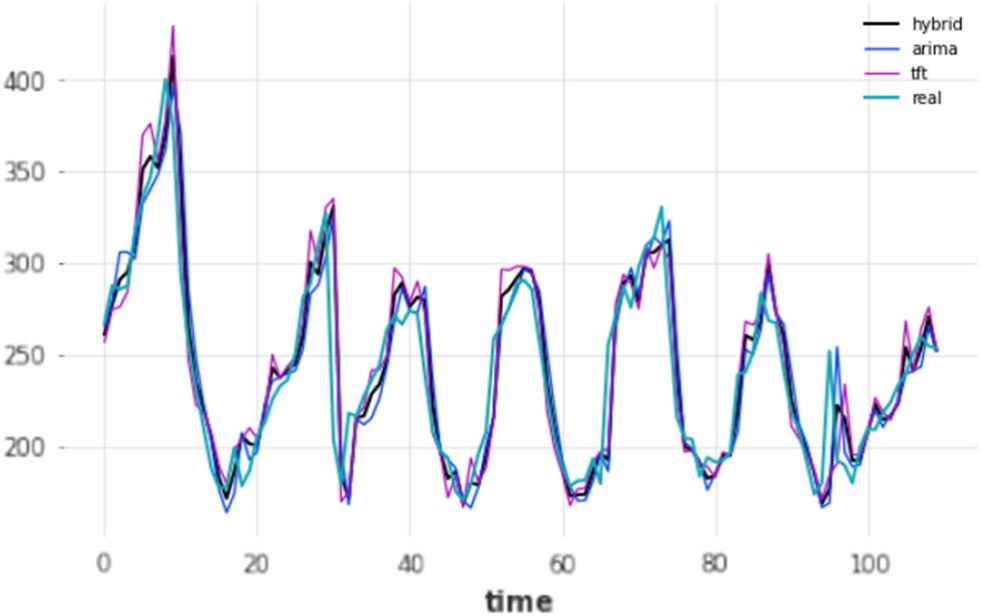
Short-term (1 h) forecasts vs ground truth for test set; split: 90–10.

Models using the short-term forecasting horizon are essentially using the lowest unit of time in the dataset, after its preprocessing, which is 1 h. In this case, evaluation is straightforward; such models only produce a single forecast, 1 h into the future and this predicted value is compared against the ground truth. A total of four different experiments took place based on the train-test split (80–20 and 90–10) as well as the re-training mechanism (yes/no). The results for each split are presented in Tables 4 and 5 respectively. The effects of re-training the deep learning models were evident at such a short forecasting horizon, as in the vast majority of cases a performance improvement was observed, highlighting the importance of the knowledge of the previous hour in predicting the next hour; most notably, the TCN’s mean absolute error dropped from 37.876 to 13.8507 after implementing the retraining approach. The forecasts of the three overall-best models against the ground truth for that period are displayed in Figs.  4 and 5 for the 80–20 and 90–10 splits correspondingly.

**Table 4:** Results for short-term forecasting horizon (1 h); split: 80–20. Ranked by name ascending, MAE descending.

Model	MAE	RMSE	MAPE	Retrain
ARIMA	17.448	24.6932	7.1174	No
ARIMA	17.448	24.6932	7.1174	Yes
DeepAR	64.7228	77.2451	27.4174	Yes
DeepAR	131.7028	138.3546	55.5346	No
ES	18.8689	26.4666	7.6474	No
ES	18.8689	26.4666	7.6474	Yes
FFT	58.8585	79.6313	20.9765	No
FFT	58.8585	79.6313	20.9765	Yes
Hybrid	**14.8924**	21.8897	**6.0875**	No
Hybrid	15.3382	**21.7836**	6.2550	Yes
LSTM	22.8856	30.9246	9.1051	Yes
LSTM	41.7699	46.5224	17.0413	No
N-BEATS	20.0911	27.3967	8.0493	Yes
N-BEATS	27.8837	36.5021	10.9623	No
TCN	15.3361	22.6401	6.2671	Yes
TCN	47.6637	52.0583	19.5002	No
TFT	15.7214	22.2269	6.4413	No
TFT	17.1849	23.1732	6.9738	Yes
Theta	18.4945	26.0843	7.548	No
Theta	18.4945	26.0843	7.548	Yes
Transformer	19.8563	26.7944	8.0028	Yes
Transformer	41.826	47.7851	16.483	No

Bold means the lowest error/best performance.

**Table 5:** Results for short-term forecasting horizon (1 h); split: 90–10. Ranked by name ascending, MAE descending.

model	MAE	RMSE	MAPE	retrain
ARIMA	16.5583	25.0833	6.947	No
ARIMA	16.5583	25.0833	6.947	Yes
DeepAR	35.9894	46.7989	16.2622	No
DeepAR	54.9676	64.7337	23.6953	Yes
ES	18.2426	26.3802	7.6694	No
ES	18.2426	26.3802	7.6694	Yes
FFT	40.0957	47.9071	16.6586	No
FFT	40.0957	47.9071	16.6586	Yes
Hybrid	14.4070	**22.0454**	6.0505	Yes
Hybrid	14.8782	23.0485	6.2390	No
LSTM	18.5033	26.8	7.7944	Yes
LSTM	26.3356	32.4004	11.2312	No
N-BEATS	20.0553	28.7076	8.4444	Yes
N-BEATS	32.9029	45.9709	13.3077	No
TCN	**13.8507**	22.3402	**5.8706**	Yes
TCN	37.876	42.9418	16.4676	No
TFT	14.9456	22.6287	6.2223	Yes
TFT	15.2929	23.151	6.3806	No
Theta	17.7509	25.8706	7.4749	No
Theta	17.7509	25.8706	7.4749	Yes
Transformer	18.466	25.2346	7.7625	Yes
Transformer	31.7511	38.764	13.6757	No

Bold means the lowest error/best performance.

**Figure 6:**
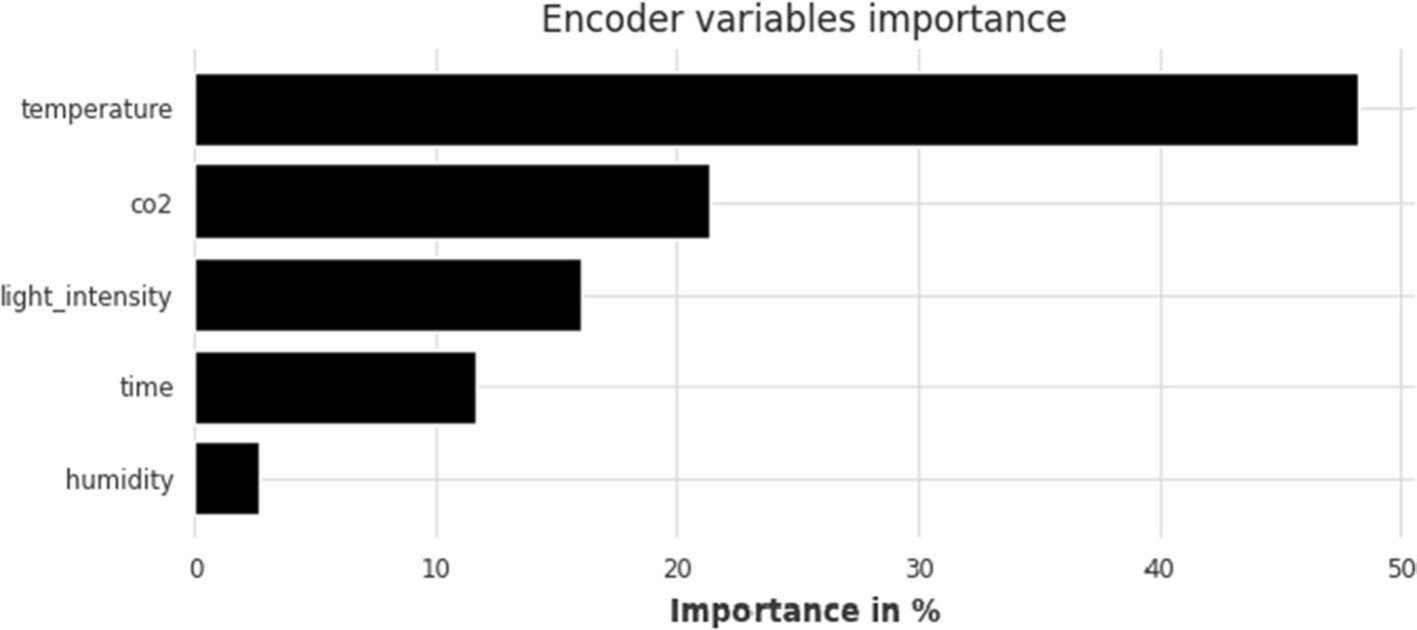
TFT feature importance for short-term forecasting horizon (1 h); split: 80–20.

In terms of interpretability, Fig. 6 displays the feature importance ranking as percentages for the TFT model when it comes to short-term forecasting. Temperature is by far the most important feature, contributing almost 50% to TFT’s CO$_2$ future forecasts. It’s followed by past values of CO$_2$ concentration, light intensity, and time, while the least important predictor of CO$_2$ is humidity. Feature importance is only displayed for the 80–20 split for all horizons; the corresponding figure for the 90–10 split is quite similar and offers no new significant insights into the model’s workings.

### Mid-term forecasting horizon

**Figure 7:**
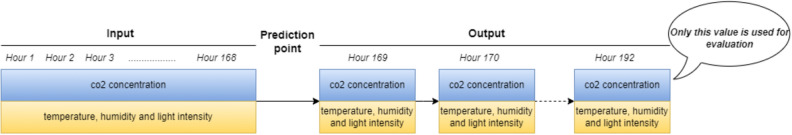
Mid-term forecasting horizon: lookback window, prediction and evaluation.

Models using the mid-forecasting horizon (24 h) have to rely on their own forecasts for the near future in order to make more forecasts for further ahead. More specifically, for each instance of the test dataset, each time-step of the forecasting horizon is calculated in an auto-regressive manner: the prediction of a time-step at a moment *t* is used for the prediction of the time-step $t+1$ for each *t* until the end of the horizon. The evaluation, however, is only based on the very last prediction value, corresponding to the very last time step of the forecasting horizon. In this case, only the prediction for the 24th h is compared against its respective ground truth. In Fig. 7, the process of making and evaluating a mid-term horizon forecast is displayed.

Similarly to the short-term horizon experiment, a total of four different settings were considered, based on the train-test split (80–20 and 90–10) as well as the re-training mechanism (yes/no). For each split, the corresponding results are presented in Tables 6 and 7 . In the mid-term horizon experiments, re-training the deep learning models with the new hourly observations, as they become known/available, did not prove as important when predicting the 24th h in the future. This was, to a high degree, expected as the correlation between the values of two successive time steps is usually much higher compared to two values being 24 time steps apart.

**Figure 8:**
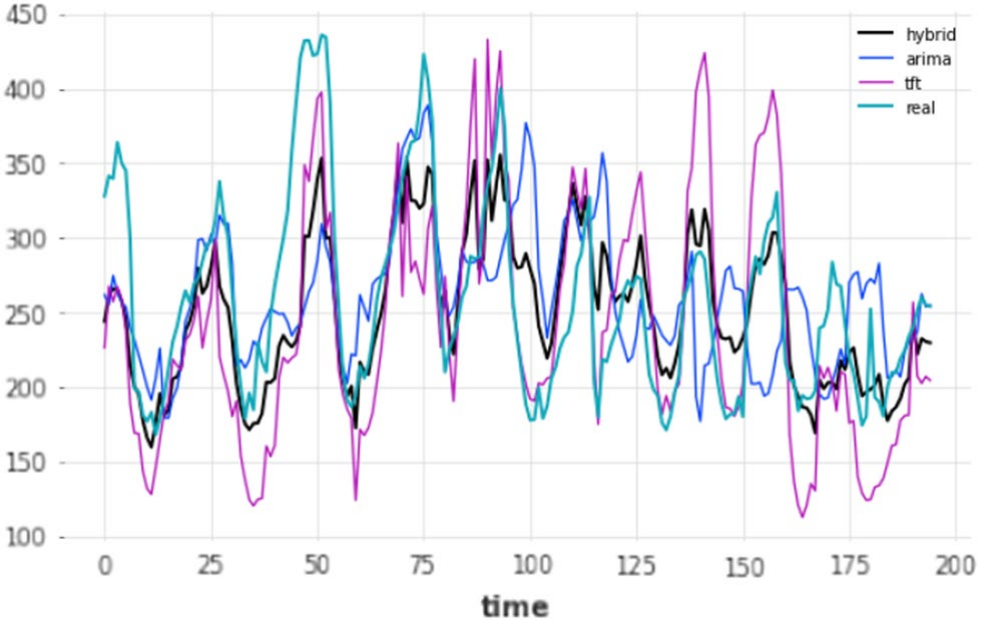
Mid-term (24 h) forecasts vs ground truth for test set; split: 80–20.

**Figure 9:**
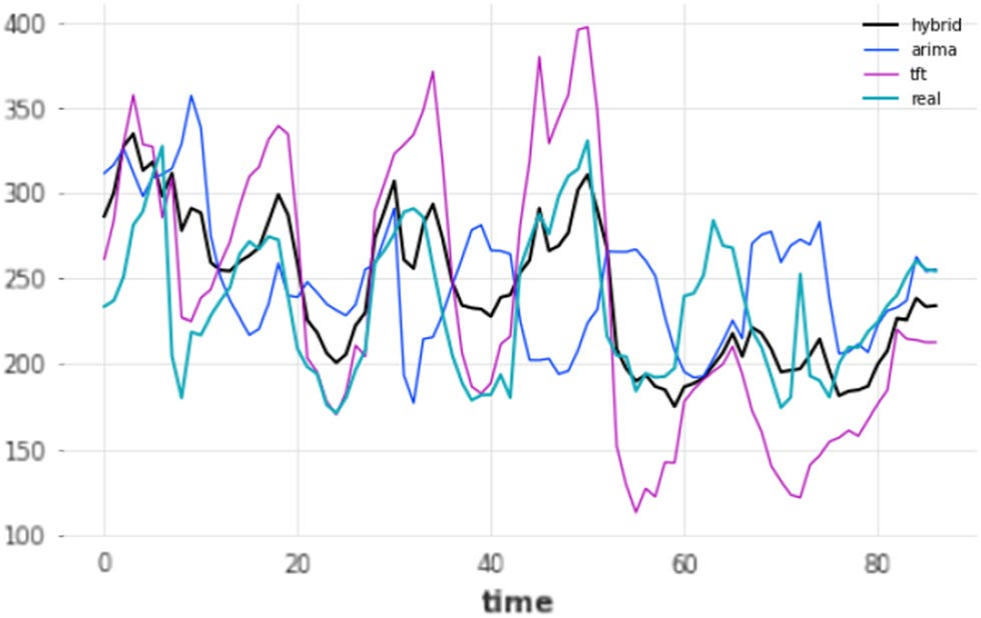
Mid-term (24 h) forecasts vs ground truth for test set; split: 90–10.

The forecasts of the three overall-best models against the ground truth for that period are displayed in Figs. 8 and 9 for the 80–20 and 90–10 splits respectively.

**Table 6:** Results for mid-term forecasting horizon (24 h); split: 80–20.

Model	MAE	RMSE	MAPE	Retrain
ARIMA	50.4179	66.5427	20.3895	No
ARIMA	50.4179	66.5427	20.3895	Yes
DeepAR	51.5311	65.1488	19.3256	Yes
DeepAR	148.1671	163.798	64.5237	No
ES	62.3673	80.3399	24.9125	No
ES	62.3673	80.3399	24.9125	Yes
FFT	78.2073	106.9273	26.0654	No
FFT	78.2073	106.9273	26.0654	Yes
Hybrid	37.2767	50.2714	14.0757	No
Hybrid	37.3014	**49.1574**	14.0701	Yes
LSTM	69.8758	88.1066	28.7614	Yes
LSTM	74.4279	90.2769	26.9331	No
N-BEATS	77.1808	94.3703	27.9407	No
N-BEATS	77.3075	95.9042	29.0755	Yes
TCN	76.0906	94.678	26.9718	No
TCN	85.9327	109.742	35.625	Yes
TFT	**36.2344**	49.7219	**13.1591**	No
TFT	51.2105	62.8617	19.2797	Yes
Theta	61.4604	79.2995	24.6313	No
Theta	61.4604	79.2995	24.6313	Yes
Transformer	67.2404	86.1076	23.2706	No
Transformer	76.9394	99.9903	32.1631	Yes

Ranked by name ascending, MAE descending. Bold means the lowest error/best performance.

**Table 7:** Results for mid-term forecasting horizon (24 h); split: 90–10.

Model	MAE	RMSE	MAPE	Retrain
ARIMA	52.0185	63.5268	23.424	No
ARIMA	52.0185	63.5268	23.424	Yes
DeepAR	35.4227	45.8422	14.8968	Yes
DeepAR	83.7181	103.7674	37.3648	No
ES	65.3607	78.0223	29.1344	No
ES	65.3607	78.0223	29.1344	Yes
FFT	42.2596	52.1752	16.9636	No
FFT	42.2596	52.1752	16.9636	Yes
Hybrid	**29.4695**	**36.5776**	**13.164**	No
Hybrid	30.7673	38.0473	13.682	Yes
LSTM	78.7371	95.8902	36.6992	No
LSTM	86.3846	106.3583	39.5714	Yes
N-BEATS	55.2179	68.9673	24.7866	No
N-BEATS	65.2014	83.7045	29.6462	Yes
TCN	58.733	70.0161	24.57	No
TCN	92.241	120.2069	42.0035	Yes
TFT	31.5093	37.5722	13.7158	No
TFT	46.5937	53.5309	19.7722	Yes
Theta	64.6421	77.2401	28.9297	No
Theta	64.6421	77.2401	28.9297	Yes
Transformer	51.3384	60.9133	21.1374	No
Transformer	78.3312	98.7249	36.9034	Yes

Ranked by name ascending, MAE descending. Bold means the lowest error/best performance.

**Figure 10:**
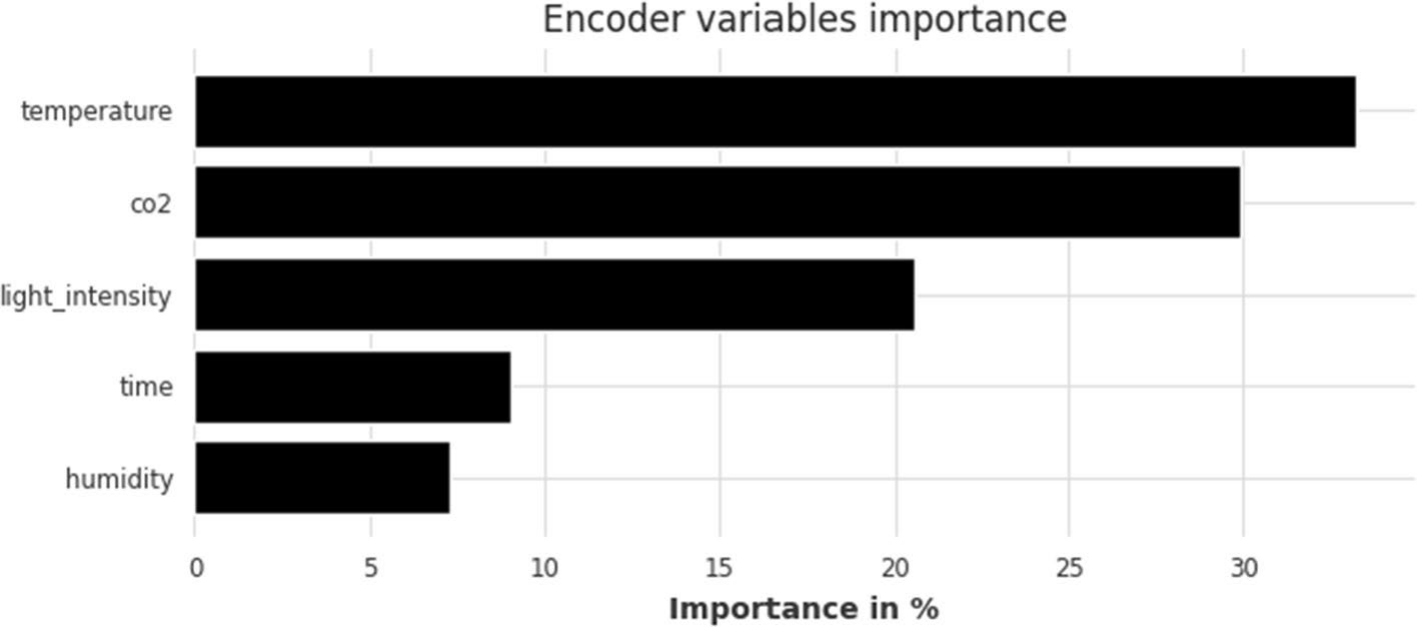
TFT feature importance for mid-term forecasting horizon (24 h); split: 80–20.

In Fig. 10 the feature importances for the TFT mid-term forecasting model are shown as percentages. In general, the trend is similar to that of short-term forecasting as the ranking of the features is exactly the same. The temperature continues to be the dominant contributing factor, however, its percentage dropped to about 35%. The importance of both CO$_2$ and light intensity increased, while on the other hand, the significance of time dropped as the horizon increased. Similarly to short-term forecasting feature importance is only displayed for the 80–20 split as the corresponding figure for the 90–10 split is quite similar and offers no additional insights into the model’s workings.

### Long-term forecasting horizon

**Figure 11:**
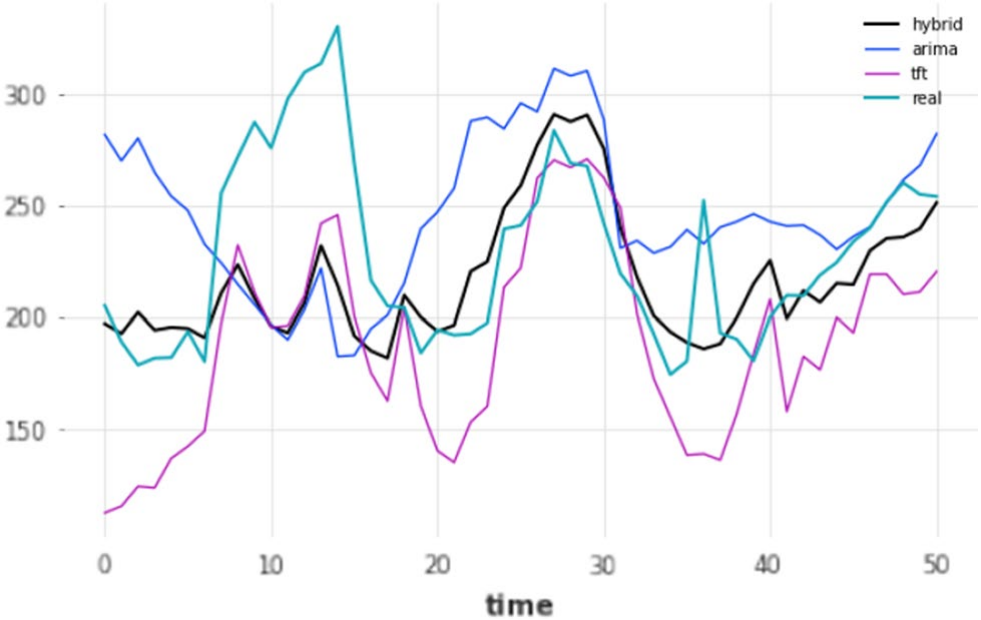
Long-term (168 h) forecasts vs ground truth for test set; split: 80–20.

Similarly to mid-term, long-term forecasting horizon models also have to rely on their own forecasts. Therefore an identical evaluation procedure was followed, where only the prediction of the very last timestamp, in this case, the 168th h, was used for evaluation. For the long-term horizon experiment, the data did not span enough into the future to perform a test using a 90–10 split. As a result, a total of two different settings were considered, based on the re-training mechanism (yes/no), but for a single split only, 80–20; results are shown in Table 8. In terms of performance, results become more unstable in general, when predicting so far into the future. This instability in forecasts is also shown in Fig.  11, where the forecasts of the three overall-best models against the ground truth for that period using an 80–20 split are displayed.

**Table 8:** Results for long-term forecasting horizon (168 h); split: 80–20.

Model	MAE	RMSE	MAPE	Retrain
ARIMA	50.0553	59.9567	22.4499	No
ARIMA	50.0553	59.9567	22.4499	Yes
DeepAR	53.6133	65.1733	21.681	No
DeepAR	361.8434	383.8009	162.3037	Yes
ES	59.5156	70.124	25.3462	No
ES	59.5156	70.124	25.3462	Yes
FFT	41.4947	57.8828	16.0627	No
FFT	41.4947	57.8828	16.0627	Yes
Hybrid	**27.60433**	**40.0019**	**11.0065**	Yes
Hybrid	52.01891	61.6153	21.8509	No
LSTM	36.3327	45.9371	14.5081	No
LSTM	47.7941	60.1755	20.4239	Yes
N-BEATS	67.8996	91.989	27.0301	Yes
N-BEATS	69.2761	87.3965	27.3918	No
TCN	41.112	53.0607	16.4957	No
TCN	57.9019	74.9861	23.097	Yes
TFT	42.7254	50.6654	18.6652	Yes
TFT	69.4283	93.8396	29.4499	No
Theta	52.4786	64.0677	22.3683	No
Theta	52.4786	64.0677	22.3683	Yes
Transformer	35.0836	44.6991	14.2247	No
Transformer	56.2405	71.9889	22.4218	Yes

Ranked by name ascending, MAE descending. Bold means the lowest error/best performance.

**Figure 12:**
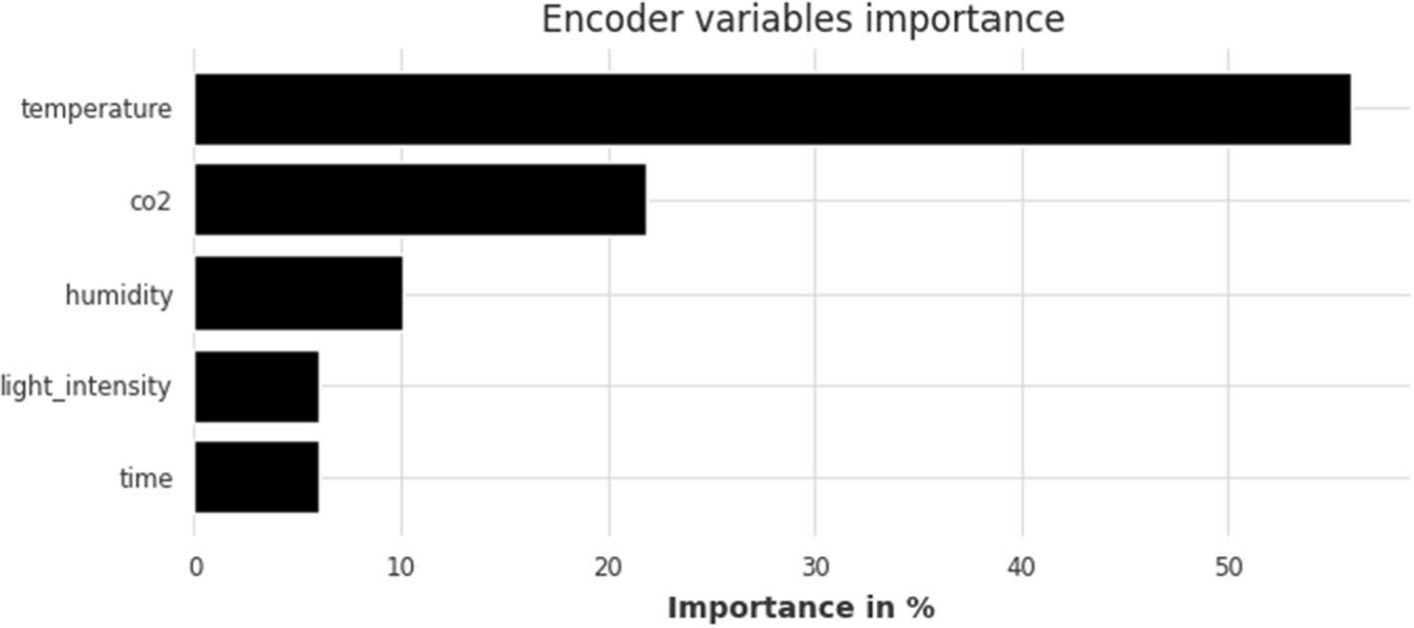
TFT feature importance for the long-term forecasting horizon (168 h); split: 80–20.

Feature importances for the long-term horizon model, shown in Fig. 12, are more or less the same as the short and mid ones. Temperature and CO$_2$ remain the two most powerful predictors and there are two notable observations 1. humidity jumped two places, from last to third; its contribution, however, is still limited at approximately 10% and 2. the contribution of time as a variable continues to decline as the length of the horizon expands. Once again, feature importance is only displayed for the 80–20 split as the corresponding figure for the 90–10 split is quite similar and does not provide any extra information about the model’s behavior.

### Friedman test and Holm post-hoc test

To get a better sense of the overall model performance and rank the different models across all the different settings, Friedman’s non-parametric statistical test^82^ was applied. The final rankings for MAE, RMSE, and MAPE can be seen in Tables 9, 10, and 11 respectively. Results indicated that the hybrid approach overall produced better results than the other approaches for all three metrics and in many cases, the difference was statistically significant, as concluded by applying Holm’s posthoc test^83^.

More specifically, when the hybrid approach was retrained with each new data point as it became available, the difference in performance was statistically significant (5% significance level) in 13 out of 20 cases, in terms of RMSE, in 5 out of 20 cases for the MAE metric, and in 6 out of 20 cases regarding MAPE, as displayed in Supplementary Tables S2, S3, and S4 respectively. As anticipated, when the retraining methodology was not applied, performance dropped and the difference in performance was statistically significant in 2 out of 20 cases for the RSME metric, 3 out of 20 for the MAE, and just 1 out of 20 regarding MAPE, as shown in Supplementary Tables S5, S6 and S7 respectively. That said, retraining does not come without a cost, as it’s a computationally, and potentially environmentally expensive procedure. It should therefore be preferred as long as the circumstances allow for it.

**Table 9:** Friedman statistical test ranking using the MAE.

Model	Ranking (MAE)
Hybrid with retraining	**2.2**
Hybrid without retraining	4.0
TFT with retraining	5.8
TFT without retraining	7.2
ARIMA without retraining	7.3
ARIMA with retraining	7.3
Theta without retraining	11.5
Theta with retraining	11.5
Transformer without retraining	12.2
TCN with retraining	13.6
TCN without retraining	14.8
LSTM without retraining	15.0
ES with retraining	15.1
ES without retraining	15.1
FFT with retraining	15.3
FFT without retraining	15.3
LSTM with retraining	15.4
DeepAR with retraining	16.4
Transformer with retraining	16.6
N-BEATS without retraining	17.6
N-BEATS with retraining	17.8
DeepAR without retraining	21.2

Bold means the lowest error/best performance.

**Table 10:** Friedman statistical test ranking using the RMSE.

Model	Ranking (RMSE)
Hybrid with retraining	**1.4**
Hybrid without retraining	4.2
TFT with retraining	4.6
TFT without retraining	7.0
ARIMA without retraining	7.5
ARIMA with retraining	7.5
Theta without retraining	12.1
Theta with retraining	12.1
Transformer without retraining	12.2
TCN with retraining	14.6
ES with retraining	14.7
ES without retraining	14.7
TCN without retraining	14.8
LSTM without retraining	14.8
DeepAR with retraining	15.6
FFT without retraining	15.7
FFT with retraining	15.7
LSTM with retraining	15.8
Transformer with retraining	16.4
N-BEATS without retraining	17.2
N-BEATS with retraining	18.2
DeepAR without retraining	21.4

Bold means the lowest error/best performance.

**Table 11:** Friedman statistical test ranking using the MAPE.

Model	Ranking (MAPE)
Hybrid with retraining	**1.8**
Hybrid without retraining	4.2
TFT with retraining	5.2
TFT without retraining	7.2
ARIMA without retraining	9.1
ARIMA with retraining	9.1
Transformer without retraining	11.0
Theta without retraining	11.5
Theta with retraining	11.5
TCN with retraining	13.8
FFT without retraining	13.9
FFT with retraining	13.9
ES with retraining	15.1
ES without retraining	15.1
TCN without retraining	15.2
LSTM without retraining	15.2
DeepAR with retraining	16.0
LSTM with retraining	16.4
Transformer with retraining	17.2
N-BEATS without retraining	17.6
N-BEATS with retraining	18.4
DeepAR without retraining	20.2

Bold means the lowest error/best performance.

## Conclusion

Accurate prediction of CO$_{2}$ levels can greatly assist data-driven decision-making around carbon emissions handling and eventually lead to its automation in the future as smart city projects develop worldwide. In this work, a hybrid system was proposed for CO$_{2}$ concentration forecasting using a multivariate time series dataset consisting of IoT sensor measurements. Furthermore, both traditional time series and deep learning models, including the current state-of-the-art architectures such as attention-based, transformer networks, were employed and compared against, for the same problem, across a series of different experiments, regarding three different forecasting horizons: short-, mid-, and long-term.

In terms of performance evaluation, the MAE, RMSE, and MAPE were measured and reported. Despite the fact that there is no one-size-fits-all solution, results indicated that, in general, deep learning architectures, capable of better exploiting the complex relationships arising in multivariate settings, tended to outperform traditional time series methods. Using Friedman’s test to produce the relative ranking of the algorithms, it was shown that the hybrid approach overall produced overall better results than the other approaches when measured across different settings. At the same time, insights were offered into the inner workings of the hybrid system’s most complex component, the Temporal Fusion Transformer, in the form of feature importance, illustrating its interpretability potential.

In the future, the proposed predictive system could be enhanced with more dedicated components aimed at improving the quality of the data, before it’s provided as input to the models. To this end, some immediate enhancements include better data imputation (how to better deal with missing data), better outlier detection and handling, as well as better feature generation through the use of embedding layers. Data quality improvements can result in substantial performance gains and promote robustness. Finally, a very crucial, but often overlooked, aspect of systems that aim to be deployed in a real-world setting, such as a smart city, is their continuous monitoring, which ensures that the corresponding system is always working as intended, the input data is up-to-date and its predictions meet certain quality standards.

### Supplementary Information

Supplementary Tables.

## Data Availability

The original dataset, as well as the necessary source code used in this study to parse and preprocess the data, train and evaluate the models, draw the graphs and create the tables, can be found at the following public GitHub repository: https://github.com/ML-Upatras/co2-concentration-forecasting. Correspondence and requests for materials should be addressed to P.L.
