# Supplementary material for: CO2 concentration forecasting in smart cities using a hybrid ARIMA–TFT model on multivariate time series IoT data
Source: Sci Rep. 2023 Oct 12;13:17266. doi: 10.1038/s41598-023-42346-0 (PMC10570338; doi:10.1038/s41598-023-42346-0)
Supplement: Supplementary file 1 — Supplementary Tables. [file 41598_2023_42346_MOESM1_ESM.pdf]

Temporal Fusion Transformer (TFT)	Number of attention heads: 4<br>Number of LSTM layers: 1<br>Number of LSTM-layer neurons: 64<br>Dropout percentage: 10%<br>Optimizer: Adam<br>Learning rate: 0.001<br>Loss function: Mean Squared Error Loss
Long Short-Term Memory Network (LSTM)	Number of hidden layers: 2<br>Number of hidden-layer neurons: 16<br>Dropout percentage: 10%<br>Optimizer: Adam<br>Learning rate: 0.001<br>Loss function: Mean Squared Error Loss
N-BEATS	Number of stacks: 10<br>Number of blocks: 1<br>Number of hidden-layers: 4<br>Number of hidden-layer neurons: 512<br>Dropout percentage: 0%<br>Optimizer: Adam<br>Learning rate: 0.001<br>Loss function: Mean Squared Error Loss
Deep AR	Number of hidden-layers: 4<br>Number of hidden-layer neurons: 20<br>Dropout percentage: 0%<br>Optimizer: Adam<br>Learning rate: 0.001<br>Loss function: Mean Squared Error Loss
Temporal Convolutional Network (TCN)	Dilation base: 2<br>Number of kernels: 3<br>Kernel size: 4<br>Dropout percentage: 10%<br>Optimizer: Adam<br>Learning rate: 0.001<br>Loss function: Mean Squared Error Loss
Transformer Network	Number of attention heads: 8<br>Number of features: 16<br>Number of encoding layers: 2<br>Number of decoding layers: 2<br>Number of feedforward layer neurons: 128<br>Dropout percentage: 0%<br>Optimizer: Adam<br>Learning rate: 0.001<br>Loss function: Mean Squared Error Loss
Exponential Smoothing	Trend: Additive<br>Seasonality: Additive
Theta	Theta: 2<br>Trend: Linear<br>Seasonality: None<br>Mode: Additive
ARIMA	p: 12<br>d: 1<br>q: 0<br>Trend: Constant
Fast Fourier Transform (FFT)	Number of frequencies: 10<br>Polynomial degree: 3<br>Trend: None

Table S1: Final set of training hyperparameters for each model

**Versus**	**Retraining**	**Statistic**	**Adjusted p-values**	**Null Hypothesis**
DeepAR	No	4.47214	0.00018	Rejected
Nbeats	Yes	3.75659	0.00379	Rejected
Nbeats	No	3.53299	0.00863	Rejected
Transformer	Yes	3.35410	0.01592	Rejected
LSTM	Yes	3.21994	0.02436	Rejected
FFT	Yes	3.19758	0.02495	Rejected
FFT	No	3.19758	0.02495	Rejected
DeepAR	Yes	3.17522	0.02495	Rejected
LSTM	No	2.99633	0.04099	Rejected
TCN	No	2.99633	0.04099	Rejected
Exponential Smoothing	No	2.97397	0.04099	Rejected
Exponential Smoothing	Yes	2.97397	0.04099	Rejected
TCN	Yes	2.95161	0.04099	Rejected
Transformer	No	2.41495	0.15737	Accepted
Theta	No	2.39259	0.15737	Accepted
Theta	Yes	2.39259	0.15737	Accepted
Arima	Yes	1.36400	0.86284	Accepted
Arima	No	1.36400	0.86284	Accepted
TFT	Yes	1.25220	0.86284	Accepted
TFT	No	0.71554	0.94855	Accepted
Hybrid	No	0.62610	0.94855	Accepted

Table S2: Holm's post-hoc test using RMSE; significance level: 5%; Hybrid with Retraining versus All.

Versus	Retraining	Statistic	Adjusted p-values	Null Hypothesis
DeepAR	No	4.24853	0.00049	Rejected
Nbeats	Yes	3.48827	0.01070	Rejected
Nbeats	No	3.44354	0.01206	Rejected
Transformer	Yes	3.21994	0.02564	Rejected
DeepAR	Yes	3.17522	0.02845	Rejected
LSTM	Yes	2.95161	0.05690	Accepted
FFT	Yes	2.92925	0.05776	Accepted
FFT	No	2.92925	0.05776	Accepted
Exponential Smoothing	No	2.88453	0.05880	Accepted
Exponential Smoothing	Yes	2.88453	0.05880	Accepted
LSTM	No	2.86217	0.05880	Accepted
TCN	No	2.81745	0.05880	Accepted
TCN	Yes	2.54912	0.11880	Accepted
Transformer	No	2.23607	0.25347	Accepted
Theta	No	2.07954	0.33811	Accepted
Theta	Yes	2.07954	0.33811	Accepted
Arima	Yes	1.14039	1.00000	Accepted
Arima	No	1.14039	1.00000	Accepted
TFT	No	1.11803	1.00000	Accepted
TFT	Yes	0.80498	1.00000	Accepted
Hybrid	No	0.40249	1.00000	Accepted

Table S3: Holm's post-hoc test using MAE; significance level: 5%; Hybrid with Retraining versus All.

**Versus**	**Retraining**	**Statistic**	**Adjusted p-values**	**Null Hypothesis**
DeepAR	No	3.95295	0.00185	Rejected
Nbeats	Yes	3.56625	0.00833	Rejected
Nbeats	No	3.39438	0.01513	Rejected
Transformer	Yes	3.30845	0.01970	Rejected
LSTM	Yes	3.13658	0.03419	Rejected
DeepAR	Yes	3.05065	0.04339	Rejected
LSTM	No	2.87878	0.07186	Accepted
TCN	No	2.87878	0.07186	Accepted
Exponential Smoothing	No	2.85730	0.07186	Accepted
Exponential Smoothing	Yes	2.85730	0.07186	Accepted
FFT	Yes	2.59950	0.13070	Accepted
FFT	No	2.59950	0.13070	Accepted
TCN	Yes	2.57801	0.13070	Accepted
Theta	No	2.08389	0.40887	Accepted
Theta	Yes	2.08389	0.40887	Accepted
Transformer	No	1.97648	0.43291	Accepted
Arima	Yes	1.56829	1.00000	Accepted
Arima	No	1.56829	1.00000	Accepted
TFT	No	1.16011	1.00000	Accepted
TFT	Yes	0.73044	1.00000	Accepted
Hybrid	Yes	0.51560	1.00000	Accepted

Table S4: Holm's post-hoc test using MAPE; significance level: 5%; Hybrid with Retraining versus All.

**Versus**	**Retraining**	**Statistic**	**Adjusted p-values**	**Null Hypothesis**
DeepAR	No	3.84604	0.00276	Rejected
Nbeats	Yes	3.13050	0.03839	Rejected
Nbeats	No	2.90689	0.07666	Accepted
Transformer	Yes	2.72800	0.12744	Accepted
LSTM	Yes	2.59384	0.18033	Accepted
FFT	Yes	2.57148	0.18228	Accepted
FFT	No	2.57148	0.18228	Accepted
DeepAR	Yes	2.54912	0.18228	Accepted
LSTM	No	2.37023	0.26665	Accepted
TCN	No	2.37023	0.26665	Accepted
Exponential Smoothing	No	2.34787	0.26665	Accepted
Exponential Smoothing	Yes	2.34787	0.26665	Accepted
TCN	Yes	2.32551	2.32551	Accepted
Transformer	No	1.78885	0.73638	Accepted
Theta	No	1.76649	0.73638	Accepted
Theta	Yes	1.76649	0.73638	Accepted
Arima	Yes	0.73790	1.00000	Accepted
Arima	No	0.73790	1.00000	Accepted
TFT	No	0.62610	1.00000	Accepted
Hybrid	Yes	0.62610	1.00000	Accepted
TFT	Yes	0.08944	1.00000	Accepted

Table S5: Holm's post-hoc test using RMSE; significance level: 5%; Hybrid without Retraining versus All.

Versus	Retraining	Statistic	Adjusted p-values	Null Hypothesis
DeepAR	No	3.84604	0.00276	Rejected
Nbeats	Yes	3.08577	0.04467	Rejected
Nbeats	No	3.04105	0.04951	Rejected
Transformer	Yes	2.81745	0.09681	Accepted
DeepAR	Yes	2.77272	0.10562	Accepted
LSTM	Yes	2.54912	0.19439	Accepted
FFT	Yes	2.52676	0.19571	Accepted
FFT	No	2.52676	0.19571	Accepted
Exponential Smoothing	No	2.48204	0.19595	Accepted
Exponential Smoothing	Yes	2.48204	0.19595	Accepted
LSTM	No	2.45967	0.19595	Accepted
TCN	No	2.41495	0.19595	Accepted
TCN	Yes	2.14663	0.35005	Accepted
Transformer	No	1.83358	0.66717	Accepted
Theta	No	1.67705	0.84179	Accepted
Theta	Yes	1.67705	0.84179	Accepted
Arima	Yes	0.73790	1.00000	Accepted
Arima	No	0.73790	1.00000	Accepted
TFT	No	0.71554	1.00000	Accepted
Hybrid	Yes	0.40249	1.00000	Accepted
TFT	Yes	0.40249	1.00000	Accepted

Table S6: Holm's post-hoc test using MAE; significance level: 5%; Hybrid without Retraining versus All.

Versus	Retraining	Statistic	Adjusted p-values	Null Hypothesis
DeepAR	No	3.43735	0.01410	Rejected
Nbeats	Yes	3.05065	0.05252	Accepted
Nbeats	No	2.87878	0.08783	Accepted
Transformer	Yes	2.79285	0.10972	Accepted
LSTM	Yes	2.62098	0.17535	Accepted
DeepAR	Yes	2.53505	0.21362	Accepted
LSTM	No	2.36318	0.32614	Accepted
TCN	No	2.34276	0.31812	Accepted
Exponential Smoothing	No	2.34170	0.32614	Accepted
Exponential Smoothing	Yes	2.34170	0.32614	Accepted
FFT	Yes	2.08389	0.52038	Accepted
FFT	No	2.08389	0.52038	Accepted
TCN	Yes	2.06241	0.52038	Accepted
Theta	No	1.56829	1.00000	Accepted
Theta	Yes	1.56829	1.00000	Accepted
Transformer	No	1.46087	1.00000	Accepted
Arima	Yes	1.05269	1.00000	Accepted
Arima	No	1.05269	1.00000	Accepted
TFT	No	0.64450	1.00000	Accepted
Hybrid	Yes	0.51560	1.00000	Accepted
TFT	Yes	0.21483	1.00000	Accepted

Table S7: Holm's post-hoc test using MAPE; significance level: 5%; Hybrid without Retraining versus All.
